# ANTIBIOTICS FOR APPENDICECTOMY IN CHILDREN AND ADOLESCENTS DURING THE PERIOPERATIVE PERIOD: AN INTEGRATIVE REVIEW

**DOI:** 10.1590/1984-0462/;2019;37;4;00013

**Published:** 2019-07-04

**Authors:** Fátima Maria Castelo Branco Roque, Antônio Aldo Melo, Alberto Jorge Castelo Branco Roque, Hanne Castelo Branco Roque, Thereza Maria Magalhães Moreira, Edna Maria Camelo Chaves

**Affiliations:** a Universidade Estadual do Ceará, Fortaleza, CE, Brazil.; b Universidade Federal do Ceará, Fortaleza, CE, Brazil; c Centro Universitário Christus, Fortaleza, CE, Brazil.; d Universidade de Fortaleza, Fortaleza, CE, Brazil.

**Keywords:** Appendicitis, Appendectomy, Antibacterials, Kid, Teenager, Apendicite, Apendicectomia, Antibacterianos, Criança, Adolescente

## Abstract

**Objective:**

To analyze the preoperative use of antibiotics in children and adolescents requiring appendectomy.

**Data source:**

Integrative review was performed in the MEDLINE, Latin American and Caribbean Health Sciences (LILACS) and Cochrane databases and the PubMed portal, with no time limit. The keywords used were: appendicitis, child, adolescent and antibacterial with Boolean AND. The articles included were published in Portuguese, English or Spanish and whose participants were under 18 years of age. Review articles and guidelines were excluded. The studies were classified according to their level of evidence and 24 papers were selected.

**Data collection and analysis:**

Seven randomized clinical trial studies (level of evidence II), eight cohorts (level III), seven retrospective observational studies (level V) and two historical documentary analysis (level IV) were selected. The studies addressed antibiotics used in acute appendicitis in both uncomplicated and complicated cases. Antibiotics initiated in the preoperative period showed a decrease in the rates of surgical wound infections. First-line (empiric) regimens were tested for sensitivity to microorganisms in peritoneal material cultures, however the results were controversial. Broad-spectrum antibiotics have been suggested in some studies because they have good coverage, but in others they have not been recommended because of the risk of developing bacterial resistance. Shorter administration time and earlier change to the oral route reduced hospitalization time.

**Conclusions:**

There are several clinical protocols with different antibiotics. However, there is no standardization concerning the type of antibiotic drug, time of use, or route.

## RESUMO

**Objetivo:**

Analisar o uso de antibióticos em crianças e adolescentes no perioperatório de apendicectomia.

**Fonte de dados:**

Realizou-se uma revisão integrativa, nas bases de dados MEDLINE, Literatura Latino-Americana e do Caribe em Ciências da Saúde (LILACS) e Cochrane e no portal PubMed, sem limite de tempo. As palavras-chave utilizadas foram: apendicite, criança, adolescente e antibacterianos com booleano AND. Os artigos incluídos foram publicados nos idiomas português, inglês ou espanhol e cujos participantes tivessem idade inferior a 18 anos. Os artigos de revisão e diretrizes foram excluídos. A qualidade da evidência foi analisada, e foram selecionados 24 artigos.

**Síntese dos dados:**

Sobre os estudos selecionados, sete foram ensaios clínicos randomizados (nível de evidência II), oito coortes (nível III), sete observacionais retrospectivos (nível V) e duas análises documentais históricas (nível IV). Os estudos abordaram antibióticos usados na apendicite aguda em suas formas não complicada e complicada. Os antibióticos iniciados no pré-operatório evidenciaram diminuição nas taxas de infecção da ferida cirúrgica. Os esquemas de primeira linha (empíricos) foram testados em relação à sensibilidade dos microrganismos nas culturas de material peritoneal, no entanto os resultados foram controversos. Sugeriram-se antibióticos de amplo espectro em alguns estudos por apresentar boa cobertura, no entanto em outros eles não foram recomendados, pelo risco de desenvolver resistência bacteriana. O menor tempo de administração e a mudança mais precoce para a via oral reduziram o tempo de internação.

**Conclusões:**

Existe um grande número de protocolos clínicos com antibióticos diversos, no entanto não existe padronização em relação ao tipo de antibiótico, tempo de uso nem via.

## INTRODUCTION

Acute appendicitis is the predominant abdominal surgical emergency among children and adolescents between 10 and 20 years of age,^1^ however, its diagnosis remains a challenge for pediatricians, since the disease often manifests itself atypically, appearing as an another condition, which can delay diagnosis, which complicates the evolution of the disease, culminating in infection, perforation and sepsis, contributing to an increase in the associated morbidity rate.^2^ ^,^ ^3^

In view of the relevance of this condition, the following must be considered: early diagnosis, so as not to delay surgical intervention; and antibiotic therapy in the perioperative period, which greatly reduces the incidence of persistent or recurrent infection and can be performed with therapy according to the type of appendicitis.^4^ There are many controversies regarding prophylaxis and the treatment of acute appendicitis, in particularly to the antibiotic regimens used in Pediatric services.

It should be remembered that patients with complicated or perforated appendicitis (defined by intraoperative and / or histopathological diagnosis of perforated appendix) are more prone to the formation of intra-abdominal abscesses than those with uncomplicated appendicitis (without evidence of appendiceal perforation), and require antibiotic coverage against gram-negative and anaerobic agents. This circumstance is also valid for the prophylaxis of surgical site infections.^4^ In view of this context, it is questioned: which antibiotics have been used in the perioperative period in children and adolescents submitted to appendectomy?

This study aimed to analyze the available evidence in the literature on the use of antibiotics in children and adolescents in the perioperative appendectomy.

## METHOD

An integrative literature review was performed with six phases:

- Forming the guiding question.
- Literature search or sampling.
- Selection of the component searches of the review sample.
- Critical analysis of included studies.
- Discussion of results.
- Presentation of the review, with consequent critical examination of results.^5^

The guiding question was: what are the antibiotics used in children and adolescents in the perioperative appendectomy? We used the PICO strategy, an acronym in the English and Portuguese languages which corresponds to the following elements:

- P - population: children and adolescents undergoing appendectomy due to acute appendicitis.
- I - intervention: normalization of the use of antibiotics in the perioperative period.
- C - comparison: with patients, prior to standardization.
- O - outcomes: reduction of length of hospital stay.^6^

A matched search was conducted in the MEDLINE, Latin American and Caribbean Health Sciences (LILACS) and Cochrane databases and in the PubMed portal, without temporal delimitation of the publications, by two individual researchers, in September 2017. We used the Health Sciences Descriptors (DeCS) and Medical Subject Headings (MeSH) *apendicite*/appendicitis/*criança*/child/adolescente/adolescent/apendicectomia/appendectomy/*antibacterianos*/anti-bacterial agents. For the systematization of searches, the descriptors had to be cross-linked, using the Boolean operator AND in the following search equation: appendicitis and child and adolescent and anti-bacterial agents; appendectomy and child and adolescent and anti-bacterial agents.

The included inclusion criteria were: studies on the theme available in full, published in the Portuguese, English or Spanish languages, and whose participants were under the age of 18. The review studies and guidelines were excluded. The articles were selected in September 2017, by two researchers, in different searches. 515 articles were identified in PubMed; 339 in MEDLINE; 17 in LILACS; and 36 in Cochrane. However, according to the researchers’ agreement, only 389 articles were selected as they met the inclusion criteria. Among these 389 articles, only 106 were applicable for eligibility evaluation, because the others did not respond to the guiding question. Only 24 articles were included in the systematic review, considering that the others did not meet the age criterion or were duplicates.

**Figure 1:**
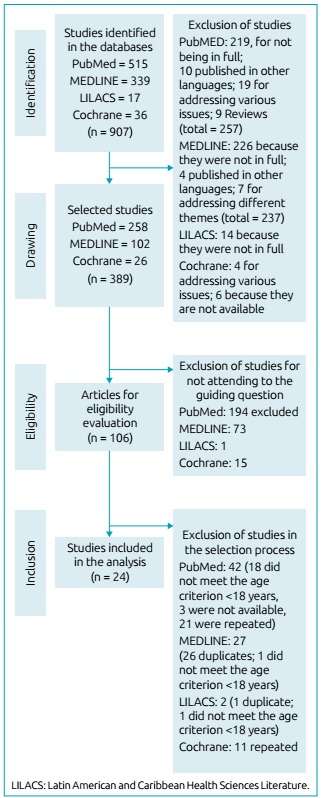
Research flowchart: identification, screening, eligibility and inclusion of scientific articles in the integrative review, according to Preferred Reporting Items for Systematic Review and Meta-Analyzes (PRISMA, 2009).^7^

In this study, we used the Preferred Reporting Items for Systematic Review and Meta-Analyzes (PRISMA)^7^ was used to explain the search and selection of studies, according to the flowchart detailed in Figure 1. The articles were classified in relation to the level of evidence (LoE) according to the adaptation of the classification proposed by Torres-Gomes.^8^ Systematic reviews of randomized clinical trials were defined as LoE I; randomized controlled trials, LoE II; cohort studies and case control, LoE III; case series, LoE IV; and narrative review, as well as other drawings, LoE V.

Subsequently, a critical and detailed analysis was carried out, with analogy to the theoretical knowledge and identification of the conclusions and implications of the standardization of the use of antibiotics in children and adolescents in the perioperative period of appendectomies. From the 24 articles selected for the literature review, two matrices were generated for the presentation of the results and discussion, seeking to integrate these for the construction of a general conception, as recommended in the literature.^5^ The first matrix shows the characterization of the studies. The second one describes the standardization used in the perioperative appendectomy and its main results.

## RESULTS

The 24 studies were coded from E1 to E24. In relation to their characterization, they present diversity in the countries where they were performed, the participants and the methodological design. These characteristics are shown in Table 1. It can be seen in Table 1 that the articles were developed in several countries, such as the United States of America (USA, E5, E7, E14, E15, E17, E19, E23, E24) , France (E2, E3, E12), England (E1, E10), Turkey (E8, E20), China (E6), New Zealand (E4), The Netherlands (E11), Ireland (E18) and Finland (E21). Such approaches were all carried out in hospitals.

**Table 1:** Characterization of the scientific production on the repercussions of the standardization of antibiotic use during the perioperative period in children and adolescents submitted to appendectomy.

Article	Authors/year/country/setting	Study design(n)
E1^9^	Wright (1982)/England/Royal Newcastle Hospital	Cohort (n=118)
E2^13^	Schmitt et al. (2012)/France/Strasbourg University Hospital	Retrospective (n=176)
E3^17^	Guillet Caruba et al. (2011)/France/Necker-Enfants Malades Hospital	Cohort (n=93)
E4^14^	Yu et at. (2014)/New Zealand/Starship Children’s Hospital	Cohort (n=47)
E5^15^	Loux et al. (2016)/United State of America/Miami Children’s Hospital	Coohort(n=115)
E6^18^	Chan et al. (2010)/China/Prince of Wales Hospital Hong Kong	Observational retrospective (n=250)
E7^10^	Kronman et al. (2016)/United State of America/ multicenter (23 independent children’s hospitals)	Retrospective cohort (n=24.984)
E8^11^	Kizilcan et al. (1992)/Turkey/Hacettepe Children’s Hospital	Randomized clinical trial (n=100)
E9^19^	Shandling et al. (1974)/Canada/Hospital for Sick Children	Retrospective(n=550)
E10^20^	Foster et al. (1987)/England/University Hospital	Randomized clinical trial (n=100)
E11^21^	Wijck et al. (2010)/Holanda/two teaching hospitals	Observational retrospective (n=49)
E12^12^	Söderquist Elinder et al. (1995)/France/St. Goran’s Children’s Hospital	Randomized clinical trial (n=544)
E13^22^	David et al. (1982)/Canada/Hospital for Sick Children	Retrospective (n=300)
E14^23^	Rice et al. (2001)/United States of America/multicenter (five centers)	Randomized clinical trial (n=26)
E15^16^	St Peter et al. (2008)/United States of America/The Children’s Mercy Hospital	Randomized clinical trial (n=100)
E16^24^	Ein et al. (2006)/Canada/Hospital for Sick Children	Cohort (n=453)
E17^25^	Fallon et al. (2011)/United States of America /Texas Children’s Hospital	Cohort(n=50)
E18^26^	Obinwa et al. (2014)/Ireland/Portiuncula Hospital	Retrospective(n=69)
E19^27^	Marchildon et al. (1977)/United States of America /Children’s Hospital of Los Angeles	Retrospective(n=89)
E20^28^	Dalgic et al. (2014)/Turkey/Sisli Etfal Training and Research Hospital	Randomized clinical trial (n=107)
E21^29^	Uhari et al. (1992)/Finland/Department of Pediatrics, University of Oulu	Randomized clinical trial (n=218)
E22^30^	Ein et al. (2013)/Canada/Hospital for Sick Children	Epidemiological - historical series (n=496)
E23^31^	Acken et at. (2016)/United States of America/Children’s Hospital Colorado	Epidemiological - historical series (n=325)
E24^32^	Desai et al. (2015)/United States of America/ Children’s Mercy Hospital and Clinics	Cohort (n=540)

Regarding the methodological design, seven studies were randomized clinical trials (LoE II), eight cohorts (LoE III), seven retrospective observational studies (LoEV) and two could be classified as documentary studies (LoE IV). Two of the studies were multicenter and performed in the United States. Regarding the sample size, samples ranging from 26 to 24,984 participants are found in the studies.

Table 2 shows the results of the randomized clinical trials, Table 3 shows the results of the cohort studies and Table 4 shows the results of the retrospective studies, with the standardization of the antibiotics used in the perioperative period in patients submitted to appendectomy. In these tables, it is possible to observe the diverse antimicrobial regimens used in the perioperative period of children and adolescents submitted to appendectomy, regarding the choice of antibiotics, associations, dose, duration of treatment and route of administration, however, the common objective was to cover aerobic (mainly gram-negative) and anaerobic microorganisms, with the knowledge that both surgical wound infection and intra-abdominal abscess formation are associated with advanced disease. First-line protocols were initiated empirically, and in cases of perforated appendicitis treatment failure, according to some studies, the result of cultures of peritoneal material collected at the time of surgery should be used in order to improve practice.

**Table 2:** Review of the main results from the randomized clinical trials.

Study	Results
E8^11^	The use of prophylactic antibiotics (ornidazole, penicillin+tobramycin and piperacillin) in uncomplicated appendicitis did not show better results than the placebo.
E10^20^	There was no difference in the surgical wound infection rates between the ampicillin / sulbactam group and those who received cefotaxime+metronidazole, therefore ampicillin / sulbactam appeared to be adequate for this prophylaxis.
E12^12^	A single dose of metronidazole in preoperative uncomplicated appendicitis in children significantly decreased the rate of infectious complications without further improvement when cefuroxime was added.
E14^23^	Treatment equivalence was found in children with perforated appendicitis between a prolonged course of intravenous antibiotics (ampicillin+gentamicin+clindamycin - ten days) and a short course of intravenous antibiotics, followed by oral antibiotics (ampicillin+gentamicin+clindamycin, intravenous, until the return of gastrointestinal function, in three to five days, followed by amoxicillin / clavulanate+metronidazole, orally, for ten days). Early use of oral antibiotics did not increase the treatment failure rate or complications.
E15^16^	The single-dose, intravenous, five-day regimen of two drugs (ceftriaxone+metronidazole) was the most efficient and cost effective treatment in children with perforated appendicitis when compared to the traditional five-day introvenous three-drug regimen (ampicillin-6 / 6h+gentamicin-8 / 8h+clindamycin-6 / 6h)
E20^28^	Ertapenem may be useful for eliminating triple regimens (ampicillin+gentamicin+metronidazole) in perforated appendicitis in children, in addition to causing less intestinal colonization by resistant bacteria.
E21^29^	The imipenem / cilastatin combination is effective and, in some cases, a slightly cheaper alternative to tobramycin+metronidazole.

**Table 3:** Review of the main results of the cohort studies.

Study	Results
E1^9^	In the preoperative period, prophylactic drugs (ampicillin, ampicillin+kanamycin, kanamycin+lincomycin) were reduced in the preoperative period, reducing surgical wound infections, without intra-abdominal abscesses in pediatric patients undergoing appendectomy due to acute appendicitis.
E3^17^	Amoxicillin / clavulanate was shown to be ineffective, with 20% of anaerobic germs showing resistance to this combination. Piperacillin / tazobactam covered the most commonly found pathogens in intra-abdominal infections, such as *Pseudomona aeruginosa* and *Escherichia coli* with intermediate resistance to amoxicillin / clavulanate or ticarcillin / clavulanate and anaerobes. A third-generation cephalosporin associated with metronidazole showed no action on *P. aeruginosa.* Carbapenems are not recommended as a broad-spectrum empiric therapy. In addition, *P. aeruginosa* and Enterococci are resistant to ertapenem, imipenem and meropenem. Piperacillin / tazobactam is chosen as a first-line therapy. If there is no isolation of pseudomonas, biological samples can be taken in order to change the broad-spectrum therapy.
E4^14^	In contrast to the use of intravenous antibiotics in a fixed period of five days, the use of clinical parameters (temperature <38ºC for 24 hours, dietary tolerance, mobilization and analgesia, via oral route only) for suspension of the antibiotics reduced hospitalization time, with no apparent impairment of results in patients with perforated appendicitis.
E5^15^	Comparison between patients with transition to oral antibiotics in perforated appendicitis and those with intravenous antibiotics for at least five days showed that the oral transition (when tolerating dietary intake) decreased hospital stay significantly, while the rate of rehospitalization was similar between groups.
E7^10^	Treatment with broad-spectrum antibiotics (piperacillin / tazobactam, ticarcillin / clavulanate, ceftazidime, cefepime or carbapenem) on the day of appendectomy or on the next day was not associated with reduced readmission rates and is probably unnecessary, especially for uncomplicated appendicitis.
E16^24^	Pediatric patients who used intravenous cefoxitin as well as the powder form preoperatively in the surgical wound had a reduction in the infection rate in relation to the untreated group in the prophylaxis of surgical wound infection.
E17^25^	A significant percentage (40%) of children with perforated appendicitis presented microorganisms resistant to the first line antibiotics in their peritoneal fluid cultures, which led to the recommendation of piperacillin / tazobactam as the most effective empiric therapy for these children.
E24^32^	Children who met the discharge criteria and had normal leukocytes levels before the fifth day of antibiotics could be safely discharged without oral antibiotics after undergoing appendectomy due to perforated appendicitis.

**Table 4:** Review of the main results of the retrospective studies.

Study	Results
E2^13^	Tested empiric antibiotic therapy remained effective for enterobacteria in complicated appendicitis in children, such as amoxicillin / clavulanate or metronidazole for anaerobes, imipenem against all microorganisms and aminoglycosides, while piperacillin, vancomycin and ticarcillin / clavulanate were associated with increased resistance rate.
E6^18^	Isolated gram-positive bacteria were sensitive to penicillin, and isolated anaerobes had the same reaction to metronidazole. As for gram-negative bacteria, 99% of Escherichia coli were sensitive to cefuroxime and only 66% of them were sensitive to gentamicin, if used instead of cefuroxime. There was no bacterial growth in children with uncomplicated appendicitis, and there was a response to the triple regimen used (ampicillin, cefuroxime and metronidazole) in 100% of the cases, however 25% of the patients with complicated appendicitis did not respond to this regimen and the collection indicated the antibiotic adjustment.
E9^19^	Children with perforated appendicitis (intraoperatively, histologically or in both cases) were no longer prone to infection complications.
E11^21^	In a study conducted in two hospitals, one group of patients post appendectomy due to perforation received five days of postoperative antibiotics while the other group remained on antibiotics for five days or more until the C-reactive protein (CRP) was less than 20 mg / mL. Prolonged use of the antibiotic did not reduce intra-abdominal abscess.
E13^21^	Pediatric patients with localized perforation or generalized peritonitis treated with ampicillin+gentamicin+clindamycin had significantly fewer infections and abscesses than those treated with ampicillin and / or gentamicin.
E18^26^	The results of the isolated microorganism sensitivity to antibiotics in peritoneal fluid cultures indicated that a amoxicillin / clavulanate+gentamicin+metronidazole combination for three to five days is empiric treatment for appendicitis related peritonitis.
E19^27^	Morbidity due to perforated appendicitis in children was reduced by factors: adequate infusion of parenteral liquids and systemic antibiotics, with inclusion for anaerobes and peritoneal drainage.
E22^30^	In perforated appendicitis in children, surgical wound infection was less frequent in those with prophylactic drainage of the peritoneal wound. Intra-abdominal abscesses were less frequent in those who used subcutaneous and intravenous prophylactic antibiotics.
E23^31^	Among children with perforated appendicitis and hospital discharge, the route of administration (intravenous or oral) to give continuity to antibiotics showed no difference in complications.

## DISCUSSION

The studies included in the review dealt with antibiotic protocols both in acute appendicitis in general and specifically in its uncomplicated and complicated forms, however the greatest number of investigations involved perforated appendicitis as it is associated with increased morbidity.^3^

There were three studies reporting appendicitis in general, as well as reduction of surgical wound infection in the pediatric population, with the prophylactic use of antibiotics in the preoperative period in patients submitted to appendectomy. One of these studies was a cohort study performed in England (1982) with 118 patients who underwent appendectomy and who had confirmed histopathological appendicitis.^9^ Thus, different antibiotics were used preoperatively, according to progression of the disease:

- Less than 24 hours and no peritonitis (group I): intravenous ampicillin.
- 24 to 48 hours without peritonitis (group II): ampicillin+kanamycin intravenously.
- Above 48 hours or with peritonitis clinic (group III): kanamycin+lincomycin, intravenously.

The continuation of the antibiotics in the postoperative period was not relevant to the study. Among all the patients, only three had wound infection (2.5%) and only one of them was a wound abscess (0.8%). There were no occurrences of intra-abdominal abscesses.^10^

In a cohort study performed over a period of 26 years, a lower rate of surgical wound infections in those who received antibiotic (cefoxitin) via the intravenous route and application of the antibiotic powder in the intraoperative wound was found when compared to the group that received only the intravenous antibiotic (p=0.03).^24^

Finally, regarding the group of prophylactic studies, there is a comparison between two antibiotic regimens used in the preoperative period, which showed no difference in infection rates between those receiving ampicillin / sulbactam and those with cefotaxime+metronidazole, which appeared to be the first adequate prophylaxis regimen for wound infection associated with pediatric appendicitis.^20^

It is worth noting that studies on the bacterial flora in complicated appendicitis (material collected from the peritoneum during surgery) and its impact on empiric therapies show positive cultures for mixed and anaerobic anaerobes, with Escherichia coli, Milleri group Streptococcus and Pseudomonas aeruginosa appearing more often.^17^ ^,^ ^18^ One of these studies demonstrated resistance to amoxicillin / clavulanate, but sensitivity to piperacillin / tazobactam in complicated appendicitis, in addition to the evidence that third generation cephalosporin + metronidazole does not include P. aeruginosa in its spectrum and that carbapenems, despite their good action, are not recommended as broad-spectrum antibiotics for empiric therapy, in order to avoid the risk of emergence of bacterial resistance. Aside from that, P. aeruginosa and enterococci are usually resistant to ertapenem, imipenem and meropenem.^17^

On the other hand, a retrospective study in France developed over a 20-year period, between 1989-1991, 1999-2000 and 2009-2010, showed that there was no significant increase in the resistance rates of enterobacteria in perforated appendicitis with empiric antibiotic protocols, remaining effective against this microbiota: amoxicillin+clavulanate (100% susceptibility of this compound to anaerobes); imipenem, which has remained effective against all microorganisms; metronidazole, which maintained efficient action against anaerobes (93% susceptibility), as well as aminoglycosides (greater than 90% susceptibility), while ticarcillin / clavulanate was more efficient than expected.^13^

The results show that 25% of the patients with complicated appendicitis did not respond to the triple regimen (ampicillin+cefuroxime+metronidazole), and the result of the peritoneal fluid collection guided the adjustment of the antibiotics,^18^ as the one that showed a significant percentage (40%) of patients with complicated appendicitis, presenting microorganisms resistant to first-line antibiotics (cefoxitin) and recommending piperacillin / tazobactam as the most effective empiric therapy for children with perforated appendicitis.^25^

Regarding the duration of antibiotic therapy in complicated appendicitis, the studies showed that, in contrast to a fixed period of five days, the use of clinical parameters (temperature lower than 38ºC for 24 hours, diet tolerance, independent mobilization and requiring oral analgesia only) for antibiotic suspension reduced hospitalization time without apparent impairment of results.^14^

A prospective cohort study in the United States in 2014 described the early transition from the intravenous antibiotic regimen (piperacillin / tazobactam) to oral (­metronidazole+­sulfamethoxazole / trimethoprim), with options (amoxicillin / clavulanate) for allergic patients who were tolerating the diet. The hospital stay rate was shown to be reduced, as well as readmission rates and complications, indicating a safe and effective transition for the treatment of perforated appendicitis in children.^15^

In relation to the comparison of the various antibiotic regimens, a multicenter study in the United States (in 23 independent children’s hospitals) deserves to be mentioned, which addressed appendicitis in 24,984 pediatric patients undergoing appendectomy, 17,654 (70.7%) of whom had uncomplicated appendicitis and 7,330 (29.3%) who had complicated appendicitis.

In this retrospective cohort study, broad-spectrum antibiotics (piperacillin / tazobactam, ticarcillin / clavulanate or ceftazidime or cefepime or carbapenem) were compared with narrow- spectrum antibiotics (cefoxitin or ceftriaxone+metronidazole or ceftriaxone or clindamycin+gentamicin or ampicillin / sulbactam or cefoxitin+ceftriaxone+metronidazole), with the objective of evaluating the therapeutic advantage in the empiric use of antimicrobials with broader coverage. Antibiotics were administered on the day of the appendectomy or on the following day.^10^

Regarding the results, treatment failure (postoperative infectious complication) was found in 664 patients (2.7%) in general, with 1.1% in uncomplicated appendicitis and 6.4% in complicated cases (p<0.01). Broad-spectrum antibiotic treatment was not associated with the lowest readmission rate and is probably unnecessary, especially for those with uncomplicated appendicitis.^10^

A randomized trial in the United States in 2006 also showed that the five-day intravenous regimen of two drugs with a a single daily dose (ceftriaxone+metronidazole) was the most efficient and cost-effective in children with perforated aspendicities when compared to the traditional regimen of the three drugs - ampicilina (four daily doses)+gentamicin ­(three daily ­doses)+­clindamycin (four daily doses) -, intravenously for five days.^16^

There were only two studies which focused on uncomplicated appendicitis, but they had conflicting results. The randomized clinical trial in Turkey involved 100 patients with uncomplicated acute appendicitis, and was divided into four groups:

- I: did not use antibiotics.
- II: ornidazole, antimicrobial and antiparasitic derived from 5-nitroimidazolicos, with molecular structure and pharmacological action similar to metronidazole.
- III: penicillin+tobramycin.
- IV: piperacillin.

This showed that the use of these antibiotics prophylactically gave no better results than placebos in relation to infectious post-operative complications,^11^ whereas the other study, also a randomized trial, showed that a single dose of metronidazole preoperatively significantly decreased the rate of infectious complications in children with uncomplicated appendicitis compared to the group that received no antibiotics, but no further improvement could be demonstrated when cefuroxime (against aerobic organisms) was added.^12^

In general, it was possible to see how the studies on the subject are thought-provoking and that there is a great variability with regard to the medical protocols used in the treatment of the patients submitted to the surgical procedure.

## CONCLUSION

The research found many diverse protocols for the use of antibiotics, which vary according to the severity of appendicitis. Antibiotic monotherapy, as well as narrow-spectrum antibiotics - when compared to multiple and broad-spectrum regimens - did not show any difference in infectious complication rates.

Despite the variation in choice, time of use and administration route of antibiotics, antibiotics should ensure coverage mainly against gram-negative and anaerobic microorganisms. In uncomplicated acute appendicitis, antibiotics are used prophylactically for 24 hours or less and reduce the rates of infectious postoperative complications, whereas in complicated appendicitis these drugs are used therapeutically for a period of 5‒7 days, or, according to more recent research, maintained until the clinical improvement of the patient.

Therefore, despite the evidence in the literature there is no specific conduct that can be followed. The use of complementary antimicrobial treatment in relation to appendectomy is indisputable. Thus, in order to fill this knowledge gap, further studies must be carried out on this subject in the pediatric setting, with the best possible level of evidence.
